# Personalized Mobile App–Based Program for Preparation and Recovery After Radical Prostatectomy: Initial Evidence for Improved Outcomes From a Prospective Nonrandomized Study

**DOI:** 10.2196/55429

**Published:** 2024-12-13

**Authors:** Alberto Martini, Claudia Kesch, Alae Touzani, Giorgio Calleris, Bogdan Buhas, Rawad Abou-Zahr, Razvan-George Rahota, Benjamin Pradère, Christophe Tollon, Jean-Baptiste Beauval, Guillaume Ploussard

**Affiliations:** 1 Department of Urology La Croix du Sud Hospital Quit Fonsegrives France

**Keywords:** prehabilitation, radical prostatectomy, robot, outcomes, continence, mobile app, app, electronic health, eHealth, surgical, human resource, health care, single-surgeon, implementation, betty.care app, cohort, perioperative, rehabilitation, mobile health, mHealth

## Abstract

**Background:**

eHealth can help replicate the benefits of conventional surgical prehabilitation programs and overcome organizational constraints related to human resources and health care–related costs.

**Objective:**

We aimed to assess the impact of an optimized perioperative program using a personalized mobile app designed for preparation and recovery after radical prostatectomy (RP).

**Methods:**

We report on a series of 122 consecutive robot-assisted RP before and after the implementation of the *betty.care* app (cohort A: standard of care, n=60; cohort B: optimized program, n=62). The primary end point was continence recovery, defined as “0 or 1 safety pad per day” at 6 weeks after surgery. Secondary end points were length of stay, same-day discharge, complications, readmissions, and number of days alive and out of hospital within 30 days from surgery.

**Results:**

Both cohorts were comparable in terms of age, prostate-specific antigen, prostate volume, and disease aggressiveness. Intraoperative parameters (lymph node dissection, operative time, and bilateral nerve-sparing surgery) were comparable in both groups, except for blood loss, which was significantly higher in cohort B (182 vs 125 cc; *P*=.008). The 6-week continence rate was improved in cohort B in both univariable and multivariable analyses (92% vs 75%; *P*=.01). There were trends favoring cohort B for all secondary end points with a minimal 30% benefit compared with cohort A. Grade 2 or more complications occurred less frequently in cohort B (13% vs 3.2%; *P*=.042). Same-day discharge and readmission rates were 35% and 53% (*P*=.043), and 3.3% and 1.6% (*P*=.54) in cohorts A and B, respectively. Mean length of stay was reduced by 0.2 days in cohort B (0.58 vs 0.78 days; *P*=.10). The main limitation was the absence of randomization.

**Conclusions:**

The implementation of a mobile app that provides a holistic approach to the perioperative period, integrating prehabilitation, rehabilitation, and remote monitoring, could lead to the improvement of important functional outcomes after RP and could replicate an on-site prehabilitation program. Multicenter validation is needed.

## Introduction

Despite the adoption of minimally invasive techniques, recovery after abdominal surgeries is still characterized by nonnegligible morbidity and mortality and significant health care expenditure [1,2]. Optimized perioperative pathways including enhanced recovery after surgery programs have demonstrated benefits in the oncologic surgery field by reducing hospitalization costs and perioperative complications, while maintaining suitable oncologic and functional outcomes [3-6]. Improved patient preparation and awareness to surgery, thanks to prehabilitation (PreHab) programs, helps to minimize side effects, improve patients’ postoperative psychological and physiological status, and ease postoperative recovery [7,8]. Several studies assessed the impact of these programs in patients undergoing radical prostatectomy (RP); some evidence exists on the benefits of integrating physical exercise, optimizing nutrition, and preoperative patient counseling in improving postoperative outcomes [9,10]. Previous studies on RP patients highlighted that additional efforts should focus on patient education prior to surgery in order to promote early mobilization, return to work, and to improve patients’ experience [11].

Our group demonstrated the benefits of optimizing the aforementioned pathways in the perioperative RP setting. Specifically, we found significant improvements in terms of postoperative metrics and health-related costs when a standard perioperative PreHab pathway was implemented [8,12-14]. The preoperative PreHab pathway included face-to-face workshops (pain management, bladder catheter, compression stockings, and postoperative care) and group-based seminars led by specialized nurses on diet, exercise, and physiotherapy (to reinforce pelvic floor muscles).

Such an implementation also led to meaningful improvements for patients’ functional outcomes, with a faster return to urinary continence after surgery and a shorter length of stay [13]. However, physicians may face some issues in adopting and maintaining a dedicated PreHab program, mainly because of organization constraints, financial support, and lack of health care resources. On this matter, eHealth might represent a potential solution.

Over the past decade, eHealth has emerged as a promising tool to enhance (1) physician-patient communication, (2) adherence to educational programs, and (3) treatment outcomes. eHealth can be used throughout the whole perioperative setting, that is, before and after surgery, without the need for excessive human effort and financial support [15].

Therefore, eHealth, by means of a smartphone app, could promote the worldwide spread of optimized pathways and at-home recovery by overcoming the lack of health care resources that are not uncommon in many scenarios [16].

In this study, we aimed to assess the implementation of a mobile app–based program that was made available to every patient who underwent RP at our institution. Patients were asked to join the study by their treating physician, upon urological consultation. Our ultimate goal was to reproduce and spread the benefits originating from our former on-site PreHab program which determined better postoperative outcomes in terms of continence recovery, complications, and length of stay.

## Methods

### Overview

We designed a prospective study aimed at evaluating the implementation of eHealth in the perioperative RP setting: the BETTY-RP study. Patients were offered the free of charge possibility to enroll in the study. BETTY-RP study was approved by the local ethical committee.

Patients with prostate cancer who were candidates for robotic prostatectomy were consecutively enrolled in the study between January 2023 and November 2023. The study was carried out at La Croix du Sud Hospital—a private hospital located in a suburb of Toulouse, France. The minimal follow-up for data analysis was 6 weeks after surgery. Data for cohort A were collected from January 2023 to early July 2023, when the intervention with the *betty.care* app was introduced. Data for cohort B were collected from July 2023 to November 2023.

The overall cohort included all consecutive RP patients before and after the implementation of the mobile app program.

Surgeons enrolling patients in the study complete 3 of 4 robotic operations per week. Postoperatively, all patients were managed according to the enhanced recovery after surgery protocol, as part of our standard of care [8].

In the interventional arm (cohort B), all consecutive patients had access to eHealth. The mobile app was freely downloadable on App stores or through the website: https://betty.care. The app gave patients access to checklists, remote monitoring, and multiple prehabilitation and rehabilitation educational materials including a specific module for RP (*BETTY coaching*).

The patient app included checklists before key moments (anesthesiologist visit, admission, discharge, and postoperative visit), alerts for starting or stopping activities (medications, physical activity, compressive stockings, diet, and work), and educational materials (podcasts, videos on physical activities, and articles) for improving patient information and his condition before surgery including physiotherapy exercises tailored to RP patients. Advice on a home-based, moderate-intensity exercise regimen before surgery was provided, and patients were asked to perform presurgical pelvic floor exercises. Advice on walking programs, aerobic training, and cardiorespiratory fitness was also provided in order to improve preoperative patient condition. Dietetic counseling on weight loss and oral nutrition was also given. ePatient-Reported Outcomes and Experience Measurements were prospectively collected to record patient satisfaction, experience, and specific outcomes related to RP, such as continence and sexual function (through validated questionnaires).

The patient app communicated with a surgeon app (Betty pro App). Thereby, the surgeon could check at any time within his app the patient characteristics (medical history, identity, medications, and allergies) and could follow him daily via remote monitoring with a possibility of activating thresholds alerts.

The surgeon could also use an in-app easy-to-use risk stratification tool that assessed the individual risk of complication for his patients and allowed them to better adapt the postoperative surveillance phase [17]. Postoperative course was comparable and standardized for both arms. The RP technique and the patient workup and follow-up did not change during the study period. Thromboprophylaxis was given for 2 weeks. Analgesic (acetaminophen) was prescribed on demand. Bladder catheter was removed at day 7 without previous cystogram. Postoperative visits were scheduled at 6 weeks and 6 months after RP. In case of persistent urinary incontinence defined as >1 daily pad at 6 weeks after surgery, 20 sessions (2 per week) of physiotherapist-guided pelvic floor muscle training were prescribed.

Data were collected prospectively by a research assistant (LC) for both study cohorts. The primary end point was the continence recovery, strictly defined as no need for, or 1 safety pad per day at 6 weeks after RP. At 6 weeks patients were also asked to answer a questionnaire regarding their satisfaction with our Pre-Hab App. We also assessed outcomes at 3 months among patients who completed their 3-month follow-up. According to our previous on-site program study, we aimed at reproducing a 20% benefit in absolute value [14]. On this matter, the study was designed to demonstrate an improvement in the continence rate from 70% to 90% (a=5%; b=80%), which yielded 59 patients per group. A few patients more per group were allowed to account for potential dropout.

Secondary end points were length of stay, same-day discharge, complication, readmission, and number of days alive and out of hospital within 30 days after surgery. Perioperative complications were reported according to the Clavien-Dindo classification [18]. Comparisons between cohorts A and B were made according to the use of the optimized program in univariable and multivariable models. Parameters were compared using 2-tailed tests as appropriate. Univariate and multivariate logistic regression analyses were performed to compare outcomes. The limit of statistical significance was defined as *P*<.05. SPSS (version 22.0; IBM Corp) software was used for analysis.

### Ethical Considerations

The study was approved by the institutional review board (IRB; National Ethics Committee “Ramsay santé Recherche et Enseignement” [IORG number: IORG0009085]; IRB number: IRB00010835/2023-11-004). This study was also approved by the local ethics committee of the La Croix du Sud Hospital and was conducted in line with the principles of the Declaration of Helsinki. All patients provided informed consent to participate to the study. Data were collected in a deidentified manner. No financial incentive was provided to patients to take part to the study.

## Results

Overall, 62 consecutive patients (cohort B) were enrolled in the BETTY-RP study. Their data were compared with those of 60 patients (cohort A) who were operated prior to the implementation of our eHealth program. Mean age, preoperative prostate-specific antigen, prostate volume, estimated intraoperative blood loss, and operating time were comparable between the 2 groups, as shown in Table 1. The proportion of more advanced disease, in terms of ISUP3 and pT3 disease, did not differ between both groups.

**Table 1:** Baseline characteristics of the overall cohort (comparisons according to the standard of care vs optimized pathway status).

			Cohort A		Cohort B		*P* value	
			SOC^a^ (n=60)		Optimized pathway (n=62)			
Age (years)			65		64		.60	
PSA^b^ (ng/mL)			8.5		9.4		.40	
Prostate volume (cc)			41		46		.30	
MRI^c^ lesion diameter (mm)			14		13		.80	
Grade group: ≥3, n (%)			23 (38)		27 (44)		.80	
**pT^d^ stage, n (%)**								.90
	pT3a	19 (32)		19 (31)				
	pT3b	4 (7)		3 (5)				
LND^e^, n (%)			31 (52)		33 (53)		.90	
EBL^f^ (mL)			125		182		.008	
Operative time (minutes)			80		86		.10	
LOS^g^ (days)			0.78		0.58		.10	
Bilateral NSS^h^, n (%)			52 (87)		53 (86)		.90	
Readmission, n (%)			2 (3.3)		1 (1.6)		.50	
SDD^i^, n (%)			21 (35)		33 (53)		.043	
Complications, n (%)			8 (13)		2 (3.2)		.042	
6-week continence, n (%)			45 (75)		57 (92)		.01	

^a^SOC: standard of care.

^b^PSA: prostate-specific antigen.

^c^MRI: magnetic resonance imaging.

^d^pT: pathologic T stage.

^e^LND: lymph node dissection.

^f^EBL: estimated blood loss.

^g^LOS: length of stay.

^h^NSS: nerve-sparing surgery.

^i^SDD: same day discharge.

No difference in terms of intraoperative parameters was observed, except for blood loss which was significantly higher in cohort B (182 vs 125 cc; *P*=.008). Lymph node dissection was performed in 31 (52%) and 33 (53%) of cohorts A and B, respectively (*P*=.90). Operative time was comparable in both cohorts (80 and 86 minutes in cohorts A and B, respectively; *P*=.10). Bilateral nerve-sparing surgery (NSS) was performed in 52 (87%) and 53 (86%) of cohorts A and B patients, respectively (*P*=.90).

Regarding the primary end point, the 6-week continence rate was significantly improved in cohort B relative to cohort A (57, 92% vs 45, 75%; *P*=.01). For the subgroup of patients with a >3-month follow-up, this difference persisted over time (100% vs 95.0% in cohorts B and A; *P*=.08).

There were trends favoring cohort B for all secondary end points. Grade ≥2 complications occurred less frequently in cohort B (8, 13% vs 2, 3.2%; *P*=.042). Same-day discharge and readmission rates were 35% and 53% (*P*=.043), and 3.3% and 1.6% (*P*=.50) in cohorts A and B, respectively. A minimal 30% benefit was seen in cohort B patients for all these end points. Mean length of stay was lower for cohort B (0.58 vs 0.78 days; *P*=.10).

Predictive factors for continence recovery at 6 weeks were also assessed in multivariable analyses taking into account age, optimized pathway, and NSS (Table 2). The optimized Betty pathway was found to be an independent predictive factor for better continence rates at 6 weeks (odds ratio 3.8, 95% CI 1.3-11.6; *P*=.02). Age also significantly influenced this outcome (odds ratio 0.90; 95% CI 0.83-0.99; *P*=.04).

**Table 2:** Multivariable analysis of predictors for 6-week continence rate.

	OR^a^	95% CI	*P* value
Age (years)	0.9	0.83-0.99	.04
Optimized pathway	3.8	1.26-11.56	.02
Bilateral NSS^b^	1.1	0.27-4.47	.9

^a^OR: odds ratio.

^b^NSS: nerve-sparing surgery.

The preliminary results on the use of our mobile app demonstrated a high usability and satisfaction rate (>80% at the end of follow-up, evaluated through the app).

## Discussion

### Principal Findings

In this study, we demonstrated that an optimized perioperative pathway, through a mobile app, is associated with better postoperative outcomes and could provide similar advantages that of an on-site, structured program. In fact, we found similar results, in terms of postoperative outcomes, to the ones of our earlier series where an on-site PreHab program was implemented.

Overall, our eHealth program improved surgical outcomes after RP, independently of patient-related factors and surgery refinements. Notably, ours is an expert center that already demonstrated better surgery outcomes than those reported at a nationwide level [19].

The mobile app was able to reproduce the benefits observed in our previous on-site experience in terms of length of stay, complications, readmission, and short-term functional recovery [8,14]. While previous series suggested that prehabilitation could improve patient experience and satisfaction in the perioperative setting, our study evaluated a holistic approach to surgery, integrating prehabilitation, rehabilitation, and remote monitoring. We demonstrated that our eHealth-based pathway was associated with meaningful end points such as urinary continence after RP [5-7,13,14,20]. Interestingly, our optimized pathway also translated into a wider acceptance of same day discharge which results in cost abutments at the hospital and health care levels [12,20,21].

Several factors may concur to our findings. Interventions dedicated to improving patient information may help anticipating potential issues associated with oncologic surgery or surgery in general. The preoperative time is of great importance as patients may be more receptive to modify their physiological and psychological perceptions and to capitalize on advice and physical condition improvements before surgery. In the specific setting of RP, patients also highlighted the importance of early mobilization, fast return to work, compliance in pelvic floor exercises, and the benefit of a better education, which facilitated realistic expectations of the postoperative recovery pathway [11]. Moreover, several studies have demonstrated that pretreatment patient-centered interventions improved satisfaction and reduced regrets in comparison with usual care for patients with newly diagnosed localized prostate cancer [22,23].

We have previously noted significant improvements in patient outcomes and satisfaction within our on-site program [10]. Despite these advantages for patients, surgeons, and hospitals, our experience taught us that the maintenance of this program and its diffusion to other centers could be challenging, mainly due to the motivation of care teams, the absence of financial and incentive support for such programs, and the lack of human resources across different health care systems. Indeed, we were not able to offer an optimized on-site pathway for all our patients due to organization constraints (only 26% of RP patients benefited from our program which was active from 2018 to 2022). Moreover, the COVID-19 outbreak led to the temporary discontinuation of this program for several months and resulted in a trend toward worse annual outcomes [19].

Such pitfalls could be overcome by eHealth, accessible to almost every patient in the whole perioperative setting, without the need for large human resources and financial support [16]. As an example, a recent study has proven that a perioperative combined eHealth care program delivering personalized care by use of goal attainment scaling reduced the time required to return to normal activities after major abdominal surgeries [15]. Other mobile apps also showed potential in terms of usability and changing risk behavior prior to major surgery. Nevertheless, the proven benefits in terms of functional recovery remain unclear [24,25]. The preliminary results on the use of our mobile app demonstrated a high usability and satisfaction rate (>80% at the end of follow-up through the app). These trends should be evaluated in a larger cohort.

### Limitations

First, the impact of the surgeon’s experience could not be precisely assessed in this series. However, the surgeon was beyond the learning curve at the beginning of the study, and no modification of surgical technique or patient selection was done during the study period. No end point difference was noted in this subanalysis suggesting that the optimized pathway was the main factor explaining outcomes improvements, and that there was no bias related to increased experience over time. A multivariable analysis was also performed to control for potential patient- and surgery-related confounding factors. Second, no randomization was performed, and the impact of this program was not assessed in a prospective, multisurgeon, multicenter study. This paper mainly represents a proof-of-concept study that will lead to further prospective evaluation. In further research, it could be also relevant to assess new patient-centered end points such as return to work, return to active life, patient satisfaction, and overall well-being. Finally, data were not collected on the proportion of eligible patients who were enrolled in the study, nor the proportion of those who completed the 6-week follow-up; therefore, response rate and attrition rates are unknown.

### Conclusions

The implementation of a mobile app that provides a holistic approach to the perioperative period, integrating prehabilitation, rehabilitation, and remote monitoring, could lead to the improvement of important functional outcomes after RP. The use of such an eHealth pathway is effortless and patient could potentially replace and improve an on-site PreHab program. Further studies as well as external series are necessary to confirm our findings.

## Abbreviations

IRB: institutional review board
NSS: nerve-sparing surgery
PreHab: prehabilitation
RP: radical prostatectomy

## Data Availability

The datasets used and/or analyzed during this study are available from the corresponding author on reasonable request.
